# Essential Oils of Two Subspecies of *Satureja montana* L. against Gastrointestinal Parasite *Anisakis simplex* and Acetylcholinesterase Inhibition

**DOI:** 10.3390/molecules29194640

**Published:** 2024-09-29

**Authors:** Francisco Les, Veronica Galiffa, Guillermo Cásedas, Cristina Moliner, Filippo Maggi, Víctor López, Carlota Gómez-Rincón

**Affiliations:** 1 Department of Pharmacy, Faculty of Health Sciences, Universidad San Jorge, 50830 Villanueva de Gállego, Spain; gcasedas@usj.es (G.C.); acmoliner@usj.es (C.M.); ilopez@usj.es (V.L.); cgomez@usj.es (C.G.-R.); 2 Instituto Agroalimentario de Aragón-IA2, CITA-Universidad de Zaragoza, 50013 Zaragoza, Spain; 3 Chemistry Interdiscplinary Project (ChIP) Research Center, School of Pharmacy, University of Camerino, Via Madonna delle Carceri 9/B, 62032 Camerino, Italy; veronica.galiffa@studenti.unicam.it (V.G.); filippo.maggi@unicam.it (F.M.)

**Keywords:** anisakiasis, enzyme inhibition, larvicide, penetration assay

## Abstract

The increasing presence of *Anisakis* spp. in fish is having significant implications for public health due to a rise in cases of anisakiasis. Given this situation, there is a critical need to develop new strategies to fight this parasite. *Satureja montana* L., commonly known as savory, is a plant recognized in folk medicine for its therapeutic activity, such as being antispasmodic and digestive, among other properties. The aim of this study was to assess the nematicide activity against *A. simplex* larvae of the essential oil from two varieties of *S. montana* (subsp. *montana* (SMM) and *variegata* (SMV)). The essential oils were obtained via hydro-distillation of the flowering aerial parts. In vitro assays demonstrated the complete inactivation of anisakis larvae after 24 h when exposed to both essential oils, along with a significant reduction in their penetration capacity. Moreover, both essential oils showed an inhibitory effect on acetylcholinesterase (AChE). No differences between the subspecies were observed in any of the assays. Hence, the nematicidal activity of essential oils could be attributed to their capacity to inhibit AChE. These findings suggest the potential of *S. montana* essential oil for therapeutic and food industry applications.

## 1. Introduction

*Satureja montana* L., commonly known as savory, is an aromatic perennial plant belonging to the Lamiaceae family. This family has the main center of differentiation in the Mediterranean basin in areas of the Mediterranean scrub, which are typical of rocky, calcareous, or sandy soils. Due to the presence of aromatic substances, many species of this family are used in cooking as a condiment, in perfumery, liqueur, and pharmaceuticals [1,2].

The etymology of the genus name *Satureja* is uncertain. Carlo Linnaeus (1707–1778), who is considered the father of taxonomy, in his publication “Specie Plantarum” claimed that the name *Starujea* comes from an ancient Roman word, whose Latin root “*satura*” means “satiated”, in reference to the alleged digestive properties of the juices of plants of this genus. The specific name (*montana*) means precisely “*of the mountains*” [1,2].

Savory is a small 20–50 cm deciduous shrub. It is perennial and woody at the base, with buds placed at a height between 2 and 30 cm (the herbaceous portions dry annually, and only the woody parts remain alive). The plants have an aromatic odor. The roots are secondary to the rhizome. The aerial part of the stem is erect, woody at the base, and pubescent all around. The sessile leaves along the stem are arranged in the opposite way (usually 2 to 2), and each subsequent pair is arranged at right angles to the one below. At the axils of the leaves, there is a bundle of 2–8 smaller leaves. The shape of the leaves is linear–lanceolate. The margins are bristly (especially at the base), while the surface is covered with glands. The inflorescences, similar in appearance to compound and terminal racemes, are formed by 2–3 vertical, closely spaced flowers. The leaves of the lower part of the inflorescence are somewhat longer. The flowers are briefly pedunculated [1,2].

As previously mentioned, savory belongs to the Lamiaceae family, with about 250 genera and almost 7000 species [3]. The family is divided into seven subfamilies: the genus *Satureja* is included in the Mentheae tribe belonging to the Nepetoideae subfamily. The subspecies (subsp.) *montana* and *variegata* are recognized as genuine for this species [2,3].

*S. montana* subsp. *montana* (SMM) is found in the western regions of the Alps, the Pyrenees, the Iberian Peninsula, and the Balkan Peninsula. It is a subspecies found from Tuscany to Calabria but is rare in the rest of Italy. The typical habitats for this plant are arid calcareous meadows and serpentine soils but also the garrigue and low spots. The preferred substrate is calcareous with a basic pH, with medium soil nutritional values, which must be arid [1,2,4].

*S. montana* subsp. *variegata* (SMV) is a common subspecies in the extreme northeast of Italy. It is also found in Slovenia and the Balkan Peninsula. The most suitable environments for this plant are the bare grasslands, meadows, and arid pastures from the hilly to the mountain level, even stony places, or places with variable humidity. The preferred substrate is calcareous with a basic pH and low soil nutritional values, which must be arid [1,2].

According to folk medicine, this plant has the following medicinal properties: antioxidant, antiseptic, carminative, digestive, and expectorant. The edible parts are used in infusion, and they can be also used as a condiment in many recipes [5,6,7]. The traditional use of *S. montana* has been widely reported as a safe aromatic plant in different cultures [8].

**Figure 1:**
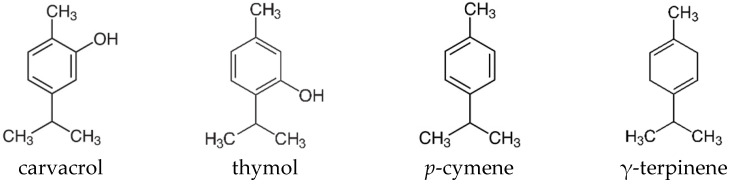
Main monoterpenes presented in SMV and SMM essential oils [2].

The composition of these two varieties has already been published by the authors, with their essential oils having the most abundant compounds, mainly monoterpenes (Figure 1) [2]. The essential oil of SMV contains a high quantity of carvacrol (22.5%), followed by thymol (17.4%), *p*-cymene (17.6%), and γ-terpinene (9.1%). The chemical composition of SMM is similar to the previous essential oil, with some differences regarding the carvacrol (61.9%) content, having a higher quantity, but being much lower for thymol (0.2%), *p*-cymene (9.9%), and γ-terpinene (8.2%). Regarding the safety of these compounds, carvacrol has been reported as generally recognized as safe (GRAS) by the Expert Panel of the Flavor and Extract Manufacturers Association (FEMA) [9].

Anisakiasis is a parasitic zoonosis due to the accidental ingestion of live nematodes belonging to the Anisakidae family, with third-stage larvae (L3), following the consumption of raw or undercooked fish [10]. The presence of the parasitic nematode in fish has recently aroused concern for consumers of fish products. The change in eating habits and the worldwide spread of “sushi” and “sashimi” or other typical dishes to be eaten raw have made anisakiasis a serious public health problem. More than 90% of cases of human infection have been recorded in Japan, reflecting the frequent habit of consuming raw fish. However, in recent years, with the development and increase in intercultural exchanges and globalization, the number of cases reported in other countries have notably increased [11].

The considerable concentration of nematodes in European fish products and the prevention against the onset of any type of infectious disease transmissible to humans by animals has led to the need for greater control. However, although the legislation in this term has addressed the issue correctly, a significant hygienic–sanitary problem has arisen in the last several years. Moreover, the ingestion or inhalation of allergens that are resistant to cooking and freezing, resulting from the presence of *Anisakis* larvae, still remain a dilemma. It is now increasingly important to encourage collaboration between operators in the sector, competent authorities, and the media in order to monitor the phenomenon and correctly inform the consumer about the actual *Anisakis* danger. Furthermore, to prevent cases of human infection and the use of chemical agents that are harmful to the environment and human health, which are used as an initial conservation treatment, attention must be paid to the development of new and safer nutrition controls and movement toward the use of products of natural origin [12].

In the literature, many studies report the nematocidal effect of some essential oils and their components [13]. For this reason, in this study, two varieties of *S. montana* have been assessed against *A. simplex* and acetylcholinesterase (AChE). The action of AChE ensures the breakdown of acetylcholine, which acts as a neurotransmitter for anisakis and other parasites [14]. Therefore, the inhibition of this enzyme is one of the main actions of organophosphates and carbamates, causing rapid muscle contractions and insect death.

## 2. Results

### 2.1. Larvicidal Activity on A. simplex

**Figure 2:**
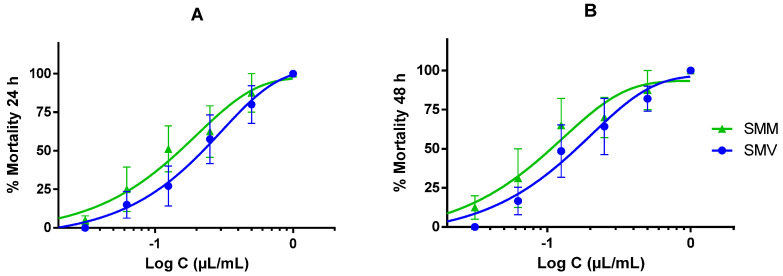
Anthelmintic activity of the *Satureja montana* essential oils. (**A**) larvicidal activity against L3 larvae of *Anisakis simplex* after 24. (**B**) activity after a treatment of 48 h.

The essential oils of *S. montana* subsp. *montana* and subsp. *variegata* showed a dose-dependent response in anisakis L3 mortality. At the maximum concentration of 1 µL/mL after 24 and 48 h, the mortality of the larvae was 100% for SMV, and the oil induced the death of all exposed larvae. At the same concentration, SMM almost achieved the same anisakis larvae mortality (Figure 2A,B). The LC_50_ of the SMM essential oil for 24 and 48 h was 151.67 and 106.34 nL/mL, and the LC_50_ of the SMV essential oil was 222.74 and 159.78 nL/mL for 24 and 48 h, respectively. No significant differences were observed between the two subsp. nor between the two periods of exposure.

### 2.2. Agar Penetration Assay

**Figure 3:**
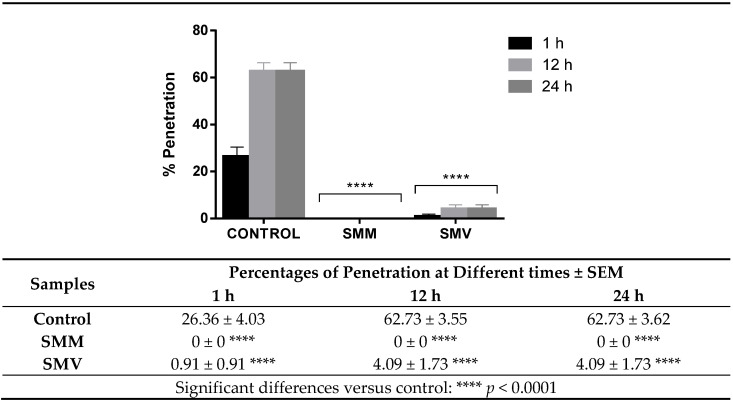
Inhibition of larval penetration after 1, 12, and 24 h of exposure to *Satureja montana* essential oils. **** *p* < 0.0001 versus control.

The larvae of *Anisakis simplex* L3 that had not been exposed to essential oils (control) and had been inserted into the 6-well microplate completely penetrated the agar block at percentages of 26.36, 62.73, and 62.73 for 1, 24, and 48 h, respectively. Parasites treated with the LC_50_ essential oils of *S. montana* subsp. *montana* and subsp. *variegata* showed an important reduction in the penetration capacity compared to the control, observing significant differences in it in all controlled periods (Figure 3).

### 2.3. Inhibition of Acetylcholinesterase

**Figure 4:**
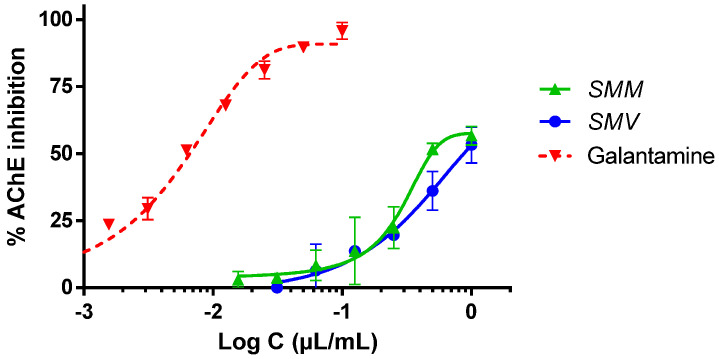
Acetylcholinesterase (AChE) inhibition by the two essential oils of *Satureja montana*. Galantamine inhibition was performed as the positive control.

The essential oils of the two *S. montana* subspecies at the concentrations evaluated showed the inhibition of AChE in a dose-dependent manner. As shown in Figure 4, at a concentration of 1 µL/mL (the highest dose tested), the inhibitions stood at 56.72 and 53.23% for the SMM and SMV essential oils, respectively. IC_50_ values were 488.88 and 872.04 nL/mL for SMM and SMV, respectively, and 6.62 nL/mL for galantamine, the reference AChE inhibitor. No significant differences were observed between the two different essential oils.

## 3. Discussion

In recent decades, various pharmaceutical, health, cosmetic, and agri-food industries have shifted toward materials with biological activity, directing the attention of the scientific community toward the development of natural products. Since ancient times, these were used in the food and cosmetics sectors not only for the antioxidant and flavoring properties but also for the antimicrobial and preservative actions, as they were able to prevent the deterioration of the products caused by pathogens [15].

The essential oils of *S. montana* subsp. *variegata* and subsp. *montana* have found use in the food sector thanks to their digestive properties and in the medical field for their antiseptic, carminative, and expectorant activities.

We demonstrated, for the first time, the nematocidal capacity against *A. simplex* L3 of the two varieties of *S. montana*. However, the acaricidal, insecticidal, and nematocidal activities of this essential oil have been widely discussed [16]. *S. montana* essential oil has previously shown nematotoxic properties in models other than *Anisakis*. In a co-cultivation model of the hairy roots of *Solanum tuberosum* L. with *Meloidogyne chitwoodi*, the addition of the essential oil of *S. montana* inhibited the increase in nematodes compared to the control [17]. This essential oil also showed a nematocidal capacity on the pine wood nematode (*Bursaphelenchus xylophilus*) [18,19]. Another study also revealed the high in vitro and in vivo anthelmintic potentials of different essential oils, including *S. montana*, against sheep gastrointestinal nematodes [20]. These properties of the essential oils tested in this study—*S. montana*, *Origanum vulgare* L., *Foeniculum vulgare* Mill., *Thymus vulgaris* L., and *S. hortensis* L.—could be due to their high contents of carvacrol, thymol, anethole, *p*-cymene, and y-terpinene.

This nematocidal activity could be explained, in part, by the anti-AChE activity. It has previously been shown that *S. montana* extracts rich in volatile compounds were capable of inhibiting AChE and butyrylcholinesterase (BChE) [21]. However, extracts with non-volatile compounds, rich in catechin, chlorogenic, vanillic, and protocatechuic acids, only partially inhibited butyrylcholinesterase and did not affect AChE.

According to the results seen in this study, no major differences are observed between the two subspecies of *S. montana* tested. However, a small trend is observed in all assays, with SMM being more potent than SMV. The chemical compositions of these essential oils of SMV and SMM are characterized by several main compounds, but in the SMV essential oil, thymol, *p*-cymene, and γ-terpinene are presented in a greater extent than in the SMM essential oil. On the contrary, there are higher percentages of carvacrol in SMM.

Previous studies showed that the AChE inhibitory effect exerted by carvacrol is 10 times stronger than that of its thymol isomer [22]. However, even more powerful than carvacrol was the activity of thymohydroquinone on this enzyme, which is present in aromatic plants of the Lamiaceae family, although it was not detected in these SMM and SMV extracts.

## 4. Materials and Methods

### 4.1. Materials and Reagents

Acetylcholinesterase (AChE) isolated from *Electrophorus electricus* (electric eel), acetylthiocholine iodide (ATCI), 5,5′-Dithiobis(2-nitrobenzoic acid) (DTNB), Tris, dimethysulphoxide (DMSO), and galantamine hydrobromide were acquired through Sigma-Aldrich (Madrid, Spain); HCl, NaCl, potassium phosphate, and MgCl_2_·6H_2_O were from Panreac (Barcelona, Spain); RPMI 1640 medium solution was from Sigma (Ronkonkoma, NY, USA); and fetal bovine serum (FBS) was from Lonza (Salisbury, MD, USA).

### 4.2. Plant Material and Essential Oils

The collection of Winter savory aerial parts was carried out in May 2014. *S. Montana* subsp. *Montana* (cultivated plants, N 45°34′46.6″, E 10°48′07.0″, 191 m a.s.l.) and *S. Montana* subsp. *Variegata* (spontaneous plants, N 45°49′06.30″, E10°57′39.34″, 678 m a.s.l.) were collected in Rivoli Veronese (Verona) and Brentonico (Trento), respectively. These plants were employed to obtain the essential oils via hydrodistillation using a Clevenger-type apparatus for 3 h. The oil yields were assessed on a dry weight basis. After collection, the oils were dried using Na_2_SO_4_ salt and stored in 4 mL vials sealed with Teflon septa at 4 °C. The chemical compositions of these essential oils are detailed in previous research [2].

### 4.3. Isolation of A. simplex Larvae and Larvicidal Activity

The *Anisakis simplex* L3 parasites were isolated from the intermediate host *Micromesistius poutassou* (blue whiting), acquired from various fish markets in Zaragoza and intended for sale. Subsequently, the larvae were placed in a sterile physiological solution containing 0.9% NaCl in order to clean them and ensure their motility [23]. Only L3 with spontaneous movement were selected, and 10 larvae were introduced for each well of a 6-well microplate containing 2 mL of sterile solution with different concentrations of the essential oil solutions to be analyzed. Parasites were incubated at 37 °C and at 5% CO_2_. After 24 and 48 h, the state of the larvae was verified, and the immobile ones were considered dead. All experiments with *Anisakis simplex* L3 larvae were performed using two plates for each concentration of essential oil and repeated three times on different days. Control wells were not exposed to the essential oils.

### 4.4. Agar Penetration Assay

In order to assess the influence of essential oils on the infective ability of nematodes, an agar penetration test was performed according to the method previously described [23,24], with some modifications. The agar solution was prepared with these reagents: 1% agar in RPMI 1640 medium solution (pH 4.0) and 20% FBS. Of this solution, 4 mL was added into every well of a 6-well microplate. The larvae of *A. simplex* L3 had previously been incubated for 1 h at 37 °C with the essential oils, with LC_50_ values of each essential oil previously calculated. Then, only the parasites considered alive were collected and washed twice with physiological 0.9% NaCl solution to eliminate external traces of essential oils. In a 6-well microplate, 10 of these parasites were placed in each well of agar exposed to the same essential oil, along with 100 µL of phosphate-buffered saline (PBS) and 1% commercial pepsin, pH 4. The control wells were not exposed to the essential oil. The parasites were incubated at 37 °C and at 5% CO_2_. After 1, 12, and 24 h, with the use of the optical microscope, the degree of penetration into agar of the larvae exposed to essential oils and of the control was observed [25].

### 4.5. Inhibition of Acetylcholinesterase

The inhibition of acetylcholinesterase (AChE) was determined with the partially modified Ellman method [26,27]. The enzyme activity was measured using a 96-well microplate, with each well containing 25 µL of a 15 mM solution of ATCI in ultrapure water, 125 µL of a 3 mM solution of DTNB in buffer C (50 mM Tris-HCl, pH 8, 0.1 M NaCl, 0.02 M MgCl_2_ 6H_2_O), 50 µL of buffer B (50 mM Tris-HCl, pH 8, 0.1% bovine serum), and 25 µL of samples. The solutions of essential oils, at different concentrations, were obtained by mixing them with the solvent (DMSO). Finally, 25 µL of AChE (0.22 U/mL) was added to every well, and the absorbance was measured 10 times every 13 s at 405 nm. Blanks and the control were measured in wells containing buffer instead of the samples or buffer instead of the enzyme, respectively. Each experiment was repeated three times and performed on different days. The positive control was assessed with galantamine, which acts as a competitive and reversible acetylcholinesterase inhibitor [28].

### 4.6. Statistical Analysis

The results were expressed as the mean ± SD of experiments assessed in triplicates. Statistical analysis of the results was performed with Graph Pad Prism v.6 software. The LC_50_ and IC_50_ were calculated using nonlinear regression and significant differences using 2-way ANOVA multiple comparisons.

## 5. Conclusions

SMM and SMV show a nematocidal capacity against *A. simplex* L3. This activity may be due to the inhibitory capacity of the enzyme AChE. These essential oils have also shown the ability to prevent the penetration of anisakis and may have potential as a preventative against this parasitosis. Although there are no significant differences between the two subspecies, SMM shows greater activity in all assays, which may be due to its richer content of carvacrol than SMV. Hence, these findings showed the promising biological effects of these two essential oil varieties. Further investigations are required in order to elucidate the mechanism of action of *S. montana* subspecies.

## Data Availability

Dataset available on request from the authors.
